# Nitrate Inhibits Nodule Nitrogen Fixation by Accumulating Ureide in Soybean Plants

**DOI:** 10.3390/plants13152045

**Published:** 2024-07-25

**Authors:** Xuelai Wang, Yuchen Zhang, Zhaohui Lian, Xiaochen Lyu, Chao Yan, Shuangshuang Yan, Zhenping Gong, Sha Li, Chunmei Ma

**Affiliations:** 1 College of Agriculture, Northeast Agricultural University, Harbin 150030, China; wangxuelai1995@163.com (X.W.); zyc18646306513@163.com (Y.Z.); 15136311538@163.com (Z.L.); xiaochenlyu@163.com (X.L.); yanchao504@neau.edu.cn (C.Y.); yssba2019@163.com (S.Y.); gzpyx2004@163.com (Z.G.); 2 College of Resources and Environment, Northeast Agricultural University, Harbin 150030, China

**Keywords:** dual-root soybean, nitrogen fixation, nitrate, asparagine, ureide

## Abstract

The mechanism by which nitrate inhibits nitrogen fixation in soybean (*Glycine max* L.) is not fully understood. Accumulation of ureide in soybean plant tissues may regulate the nitrogen fixation capacity through a feedback pathway. In this study, unilaterally nodulated dual-root soybeans prepared by grafting were grown in sand culture. They were subjected to the removal of the nodulated side roots, and were given either nitrate supply or no supply to the non-nodulated side roots for 3 days (experiment I). Additionally, they received nitrate supply to the non-nodulated side roots for 1–14 days (experiment II). The results showed that nitrate supply increased the levels of asparagine and ureide in soybean shoots (Experiment I). In Experiment II, nodule dry weight, nodule number, nodule nitrogenase activity, and nodule urate oxidase activity decreased significantly after 3, 7, and 14 days of nitrate supply. Ureide content in the shoots and nodules increased after 1, 3, and 7 days of nitrate supply, but decreased after 14 days of nitrate supply. There was a significant positive correlation between urate oxidase activity and nitrogenase activity. Hence, we deduced that nitrate supply increased the asparagine content in soybean shoots, likely inhibiting ureide degradation, which induced the accumulation of ureide in soybean shoots and nodules, and, in turn, feedback inhibited the nodule nitrogen fixation. In addition, urate oxidase activity can be used to assess the nitrogen fixation capacity of nodules.

## 1. Introduction

Nitrogen supply inhibits soybean nodule formation, growth, and nitrogen fixation [1,2,3,4]. Asparagine plays a crucial role in regulating nitrogen fixation in the nodules of legume plants [5,6]. Nitrate supply induces a very strong increase in the expression of asparagine synthetase (AS; EC 6.3.5.4) genes in soybean roots, increases the proportion of asparagine to total amino acids in xylem sap, and facilitates asparagine transport in the plant [7,8,9]. Bacanamwo and Harper (1997) found that nitrate supply to hydroponic culture of soybean led to a significant decrease in nodule nitrogenase activity and an approximately 35-fold increase in free asparagine content in the shoots, but not in the nodules, compared to the control without nitrate supply [10]. In addition, the supply of asparagine to hydroponic culture of soybean also resulted in a significant decrease in nodule nitrogenase activity, as well as an increase in asparagine and ureide content in both the shoots and nodules [11]. Lukaszewski et al. (1992) postulated that the increase in ureide content of soybean leaves caused by asparagine supply may be related to the inhibition of allantoate amidohydrolase (AAH; EC 3.5.1.5) activity, a manganese-dependent ureide-degrading enzyme. Furthermore, the mechanism underlying this inhibition is likely associated with the chelating effect between asparagine and Mn^2+^ [12]. However, Werner et al. (2008) proposed that the inhibition of allantoate amidohydrolase activity by asparagine is a competitive inhibition that is not directly related to Mn^2+^ chelation [13].

Nitrate supply to soybeans grown in hydroponic culture significantly depresses nodule nitrogen fixation activity but increases ureide concentration in plant tissues [14]. Compared with the 2 mM nitrate supply control, the 15 mM nitrate supply to soybean grown in sand significantly inhibits nitrogenase activity and causes a marked increase in ureide content in nodules. Furthermore, when nitrate is removed from all treatments, nitrogenase activity and ureide content in the nodules return to similar levels [15]. Exogenous ureides applied to soil-grown soybeans significantly decrease nitrogenase activity and significantly increase leaf ureide concentration. When exogenous ureides are removed from the soil, nitrogenase activity could recover, and leaf ureide concentration decreases to a similar level as in untreated plants [16]. Serraj et al. (1999) found that the application of exogenous ureides to hydroponically grown soybeans inhibited nodule nitrogenase activity and significantly increased shoot ureide content, but had no significant effect on nodule ureide content [17]. Vadez et al. (2000) found that the application of allantoate to hydroponically grown soybeans inhibited nodule nitrogenase activity and significantly increased nodule ureide content, but had no significant effect on shoot ureide content [11]. Serraj and Sinclair (2003) found that the supply of ureide to hydroponically grown soybeans inhibited nodule nitrogenase activity under different CO_2_ concentration conditions, and that the ureide content in plant tissues increased significantly under normal CO_2_ concentration conditions but did not change under high CO_2_ concentration conditions, suggesting that the products of ureide degradation, rather than the concentration of ureide in the plant tissues, play a crucial role in controlling nodule nitrogen fixation [18].

Most previous studies on the relationship between ureide accumulation in soybean plant tissues and nodule nitrogen fixation after nitrogen application have supplied nitrogen directly to the nodulated roots, without excluding the effect of direct contact between nitrogen and the nodule and without establishing a dynamic relationship between ureide accumulation and nitrogenase activity. In this study, we used unilaterally nodulated dual-root soybeans prepared by grafting as the experimental material and, for the nitrate supplied in the non-nodulated side roots, avoided direct contact with the nodules. Changes in the asparagine and ureide content of soybean shoots were determined under conditions where the nodulated side roots of dual-root soybeans were removed, and the non-nodulated side roots were supplied with or without nitrate. In addition, changes in nodule dry weight, nodule number, nodule nitrogenase activity, nodule urate oxidase activity, and ureide content of dual-root soybeans were determined at different times of nitrate supply to the non-nodulated side roots. The relationship between ureide content of soybean plants and nitrogen fixation of nodules under nitrate supply was studied. The aim is to provide new perspectives on the nitrate regulation of nitrogen fixation in soybean nodules.

## 2. Results

### 2.1. Effects of Nodule Nitrogen Fixation or Nitrate Supply on the Levels of Asparagine and Ureide in Dual-Root Soybean Shoots

**Figure 1:**
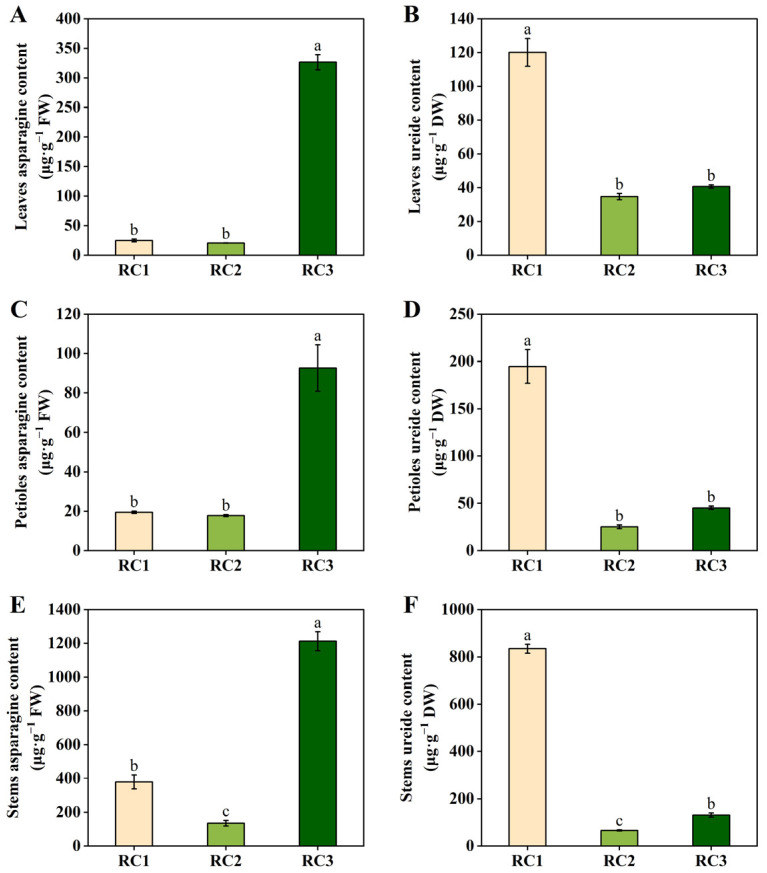
Asparagine and ureide content in leaves (**A**,**B**), petioles (**C**,**D**), and stems (**E**,**F**) after 3 days of treatments involving nodule nitrogen fixation or nitrate supply. Unilaterally nodulated dual-root soybeans prepared by grafting were used as the experimental material. In the RC1 treatment, both side roots of the dual-root soybeans were maintained and supplied with a nitrogen-free nutrient solution. In the RC2 treatment, the nodulated side roots of the dual-root soybeans were removed, while the remaining non-nodulated side roots were supplied with a nitrogen-free nutrient solution. In the RC3 treatment, the nodulated side roots of the dual-root soybeans were removed, while the remaining non-nodulated side roots were supplied with a nitrogen-containing nutrient solution at a concentration of 200 mg·L^−1^. All of these experimental treatments were conducted over a period of 3 days. The data represent means ± SE (standard error) derived from three biological replicates, each of which consists of two individual plants. Different lowercase letters indicate significant differences between treatments according to Duncan’s multiple range test (*p* < 0.05). FW, fresh weight; DW, dry weight.

Figure 1 shows the asparagine and ureide content in soybean shoots after 3 days of treatments involving nodule nitrogen fixation or nitrate supply (experiment I). The asparagine content in the leaves, petioles, and stems of the RC3 treatment was significantly higher than in those of plants having received the RC1 and RC2 treatments (Figure 1A,C,E). Evidently, compared to soybeans grown under conditions of nitrogen fixation by nodules or with a nitrogen-free supply, nitrate supply significantly increased the asparagine content in the shoots. This indicated that asparagine in the shoots is primarily derived from nitrate assimilation. The asparagine content in the leaves and petioles of the RC1 treatment showed no significant difference to the RC2 treatment. The asparagine content in the stems of the RC1 treatment was significantly higher than in those of the RC2 treatment. This indicated that, compared to a nitrogen-free supply, the asparagine content in the shoots increases under conditions of nodule nitrogen fixation.

The ureide content in the leaves, petioles, and stems of the RC1 treatment was significantly higher than in those receiving the RC2 and RC3 treatments (Figure 1B,D,F). It can be seen that soybean plants with nodule nitrogen fixation capacity have a significantly higher ureide content in the shoots than plants with a nitrogen-free supply or those supplied with nitrate. This indicates that the ureide in the shoots mainly originates from nodule nitrogen fixation. Additionally, the ureide content in the leaves and petioles of the RC3 treatment did not differ significantly from that in samples receiving the RC2 treatment. The ureide content in the stems of the RC3 treatment was significantly higher than that in samples receiving the RC2 treatment. This suggests that the nitrate had a certain degree of influence on the ureide content in the shoots.

### 2.2. Effects of Nitrate Supply on Nodulation and Nitrogenase Activity in Dual-Root Soybeans

**Figure 2:**
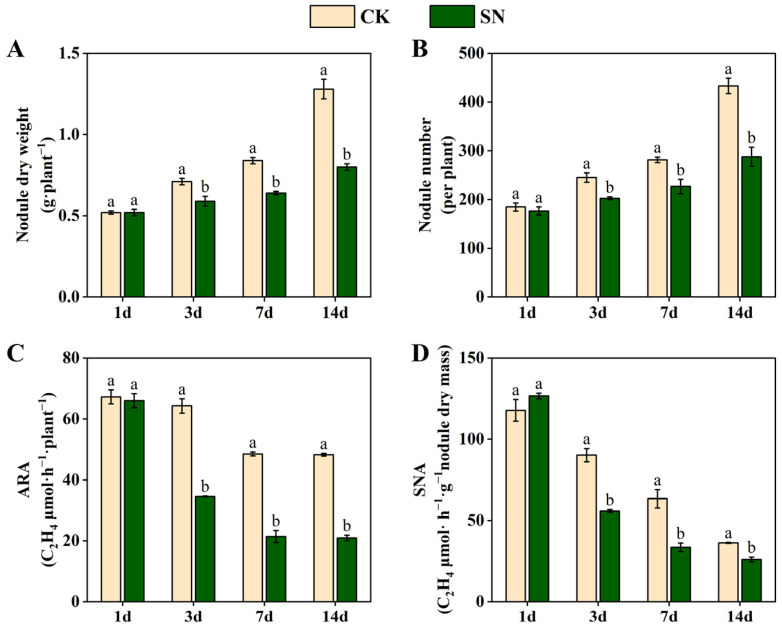
Changes in nodule dry weight (**A**), nodule number (**B**), acetylene reducing activity in μmol of ethylene formed per plant per hour (ARA) (**C**), and specific nitrogenase activity per gram dry mass of nodules per hour (SNA) (**D**) in the nodulated side roots of unilaterally nodulated dual-root soybeans. In the CK treatment, both side roots of dual-root soybeans were supplied with a nitrogen-free nutrient solution. In the SN treatment, the nodulated side roots were supplied with a nitrogen-free nutrient solution, while the non-nodulated side roots were supplied with a nitrogen-containing nutrient solution at a concentration of 200 mg·L^−1^. The data represent means ± SE (standard error) derived from three biological replicates, each of which consists of two individual plants. Different lowercase letters indicate significant differences between treatments at the same treatment time under Student’s *t*-test (*p* < 0.05).

In experiment II, the non-nodulated side roots of unilaterally nodulated dual-root soybeans were supplied with nutrient solution at two nitrogen concentrations for 1, 3, 7, and 14 days. Figure 2 shows the changes in nodule dry weight, nodule number, acetylene reducing activity in μmol of ethylene formed per plant per hour (ARA), and specific nitrogenase activity per gram dry weight of nodules per hour (SNA) of the nodulated side roots. In both the CK and SN treatments, nodule dry weight and nodule number gradually increased with increasing duration of treatment (Figure 2A,B). Compared with the CK treatment, the nodule dry weight and nodule number of the SN treatment did not change significantly after 1 day of nitrate supply, but they decreased significantly after 3, 7, and 14 days of nitrate supply. In both the CK and SN treatments, ARA and SNA gradually decreased with increasing treatment duration (Figure 2C,D). Compared with the CK treatment, the ARA and SNA of the SN treatment did not change significantly after 1 day of nitrate supply, but they decreased significantly after 3, 7, and 14 days of nitrate supply. This indicated that nitrate supplied to the non-nodulated side roots of dual-root soybeans systemically inhibits nodulation and nodule nitrogenase activity of the nodulated side roots. The inhibitory effect of nitrate on the nodule dry weight, nodule number, and ARA became more pronounced with increasing duration of treatment.

### 2.3. Effects of Nitrate Supply on Nodule Urate Oxidase Activity in Dual-Root Soybeans

**Figure 3:**
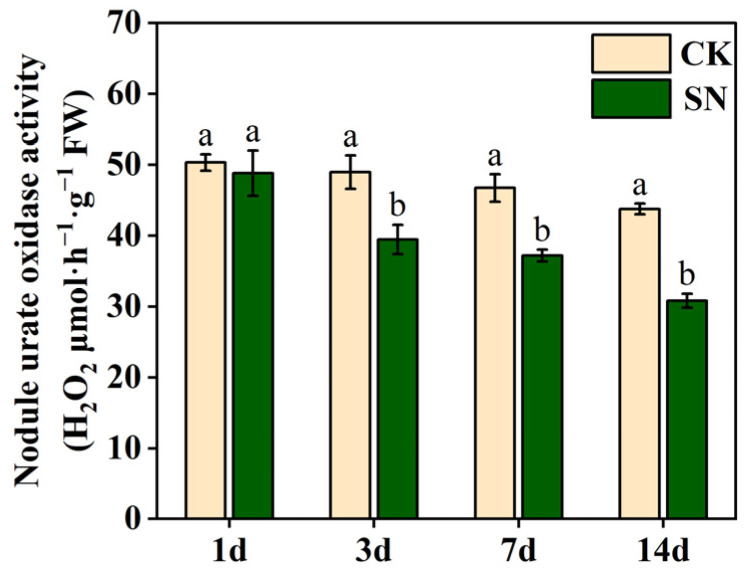
Changes in nodule urate oxidase activity in the nodulated roots of unilaterally nodulated dual-root soybeans. In the CK treatment, both side roots of unilaterally nodulated dual-root soybeans were supplied with a nitrogen-free nutrient solution. In the SN treatment, the nodulated side roots of unilaterally nodulated dual-root soybeans were supplied with a nitrogen-free nutrient solution, while the non-nodulated side roots were supplied with a nitrogen-containing nutrient solution at a concentration of 200 mg·L^−1^. The data represent means ± SE (standard error) derived from three biological replicates, each of which consists of two individual plants. Different lowercase letters indicate significant differences between treatments at the same treatment time under Student’s *t*-test (*p* < 0.05). FW, fresh weight.

Figure 3 shows the changes in nodule urate oxidase activity in the nodulated side roots of unilaterally nodulated dual-root soybeans after nitrate supply to the non-nodulated side roots for 1, 3, 7, and 14 days. In both the CK and SN treatments, nodule urate oxidase activity gradually decreased with increasing duration of treatment. Compared with the CK treatment, the urate oxidase activity of nodules exposed to the SN treatment did not change significantly after 1 day of nitrate supply, however, it decreased significantly by 19.4%, 20.4%, and 29.5% after 3, 7, and 14 days of nitrate supply, respectively. It can be seen that the magnitude of the differences between CK and SN treatments became progressively larger with longer treatment time. This indicates that nitrate supply to the non-nodulated side roots of dual-root soybeans systemically inhibits the nodule urate oxidase activity of the nodulated side roots. The inhibitory effect of nitrate on nodule urate oxidase activity became increasingly pronounced with longer treatment durations.

### 2.4. Effects of Nitrate Supply on the Levels of Ureide in Dual-Root Soybeans

**Figure 4:**
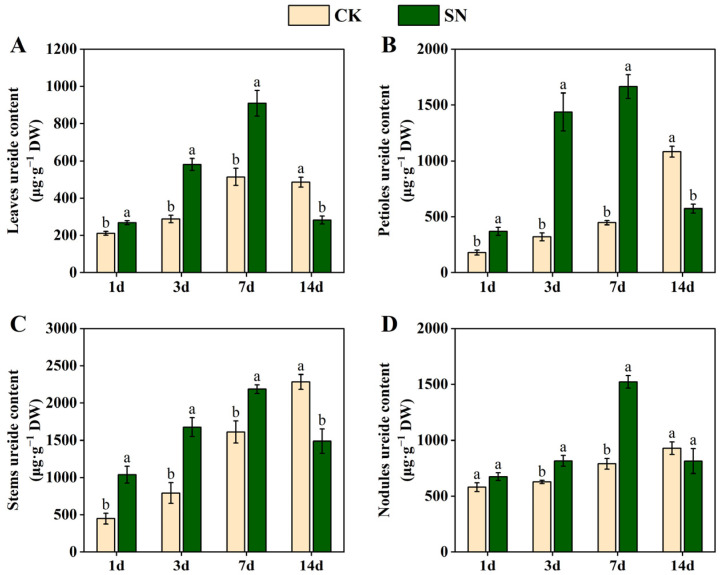
Changes in ureide content in leaves (**A**), petioles (**B**), stems (**C**), and nodules (**D**) of unilaterally nodulated dual-root soybeans. In the CK treatment, both side roots of unilaterally nodulated dual-root soybeans were supplied with a nitrogen-free nutrient solution. In the SN treatment, the nodulated side roots of unilaterally nodulated dual-root soybeans were supplied with a nitrogen-free nutrient solution, while the non-nodulated side roots were supplied with a nitrogen-containing nutrient solution at a concentration of 200 mg·L^−1^. The data represent means ± SE (standard error) derived from three biological replicates, each of which consists of two individual plants. Different lowercase letters indicate significant differences between treatments at the same treatment time under Student’s *t*-test (*p* < 0.05). DW, dry weight.

Figure 4 shows the changes in ureide content in both the shoots and nodules of unilaterally nodulated dual-root soybeans after nitrate supply to the non-nodulated side roots for 1, 3, 7, and 14 days. With increasing treatment time, the ureide content in the leaves, petioles, stems, and nodules showed a gradual increase in the CK treatment, but an initial increase and then a decrease in the SN treatment. Compared with the CK treatment, the ureide content in the leaves, petioles, and stems was significantly increased after 1, 3, and 7 days of nitrate supply, but it significantly decreased after 14 days of nitrate supply (Figure 4A–C). Changes in ureide content in the nodules were similar to those in the shoots. The ureide content of the nodules in the SN treatment was increased significantly after 3 and 7 days of nitrate supply compared with the CK treatment (Figure 4D). It was shown that nitrate supply led to an increase in ureide content in both the shoots and nodules, with a significant effect on ureide content in the shoots preceding that in the nodules. Nevertheless, a trend was observed for the ureide content in both the shoots and nodules to decrease after 14 days of nitrate supply.

### 2.5. Correlations between Urate Oxidase Activity and Nitrogenase Activity in Soybean Nodules

Table 1 shows the correlations of urate oxidase activity with ARA and SNA in soybean nodules. Urate oxidase activity exhibited a highly significant positive correlation (*p* < 0.01) with ARA and a significant positive correlation (*p* < 0.05) with SNA in nodules.

## 3. Discussion

Asparagine is an important amino acid in soybean plants, and nitrate supply enhances its synthesis and transport, leading to an increase in asparagine content in soybean plants [7,8,9]. The present study also showed that nitrate supply increased the asparagine content in soybean shoots (Figure 1A,C,E), suggesting that asparagine in the shoots is mainly derived from nitrate assimilation. The ureides allantoin and allantoate, which are major organic nitrogen compounds, are transported from soybean nodules to shoots, and, upon degradation, release ammonium that is synthesized into asparagine [18,19]. Furthermore, nodules can also synthesize asparagine directly and transport it upwards [20,21,22]. In this study, after excluding the effect of nitrate on asparagine content, we found that the content of asparagine in the shoots was higher in plants with nodules than in plants without nitrogen supply (Figure 1A,C,E), further confirming that the asparagine in the shoots can originate either from direct synthesis in soybean nodules or from the metabolism of ureide degradation. Asparagine acts as a signaling molecule that regulates nitrogen fixation in soybean nodules [5,10], and its inhibition of ureide breakdown led to ureide accumulation in plants [12,13]. This study also found that after nitrate supply both asparagine and ureide levels increased simultaneously in the shoots (Figure 1), probably due to asparagine inhibiting ureide breakdown.

Urate oxidase (UO; EC 1.7.3.3) is considered a key enzyme in the ureide synthesis pathway, catalyzing the oxidation of uric acid to allantoin [23,24,25]. In this study, the nitrate supply to the non-nodulated side roots of unilaterally nodulated dual-root soybeans significantly reduced nodule urate oxidase activity in the nodulated roots, and the reduction increased with the duration of nitrate supply (Figure 3), indicating that a continuous nitrate supply diminishes ureide synthesis in the nodules. However, the first 7 days of nitrate supply increased the ureide content in both the shoots and nodules (Figure 4), likely because of the inhibition of ureide degradation by asparagine in the shoots following nitrate supply.

After nitrogen supply to soybeans, there was a notable decrease in nodule dry weight, nodule number, and nodule nitrogenase activity [1,2,5,26]. In this study, nitrate supply to the non-nodulated side roots of unilaterally nodulated dual-root soybeans significantly reduced the nodule dry weight, nodule number, and ARA and SNA in the nodulated side roots (Figure 2). Lyu et al. (2020) established that ARA reliably reflected the nitrogen fixation capacity of soybean nodules [27]. This study revealed a consistent impact of nitrate supply to the non-nodulated side roots of unilaterally nodulated dual-root soybeans on ARA and urate oxidase activity in the nodules (Figure 2C and Figure 3). Moreover, urate oxidase activity and ARA showed a highly significant positive correlation (r^2^ = 0.953) (Table 1), suggesting that urate oxidase activity can serve as an indicator of the nitrogen fixation capacity of the nodules. Therefore, assessing urate oxidase activity could be a valuable method for evaluating the nitrogen fixation capacity of soybean nodules.

The accumulation of ureide in soybean plants has been shown to potentially regulate nitrogenase activity through a feedback inhibition pathway, as indicated by previous studies [28,29,30]. An increase in ureide and asparagine supply leads to higher ureide content in shoots and a decrease in nitrogenase activity [11,17]. In this study, providing nitrate to the non-nodulated side roots of unilaterally nodulated dual-root soybeans during 1–7 days resulted in elevated ureide content in both the shoots and nodules, while reducing nitrogenase activity in the nodules (Figure 2C,D and Figure 4). Notably, a significant increase in ureide content in the shoots was observed after only one day of nitrate supply (Figure 4A–C). However, a significant decrease in nodule nitrogenase activity was not observed until 3 days after nitrate supply (Figure 2C,D), indicating a possible inhibitory effect of ureide accumulation in the shoots on nodule nitrogenase activity. Furthermore, a significant decrease in ureide content was observed in the shoots after 14 days of nitrate supply (Figure 4). This reduction can be attributed to the accumulation of ureide in the shoots after nitrate supply, leading to feedback inhibition of nodule nitrogenase activity and subsequent attenuation of ureide synthesis in the nodules. This ultimately resulted in a reduction in ureide content in the shoots.

## 4. Materials and Methods

### 4.1. Plant Materials and Growth Conditions

Soybean plants were grown in sand culture. Seeds of a nodulated soybean variety (*Glycine max* (L.) Merr. *cv*. Heinong 40, obtained from the Heilongjiang Academy of Agricultural Sciences, Harbin, China) and a non-nodulated soybean variety (*Glycine max* (L.) Merr. *cv*. WDD01795, L8-4858, obtained from the Chinese Academy of Agricultural Sciences, Beijing, China) were germinated in fine sand in the greenhouse. Four-day-old seedlings were used for grafting to prepare unilaterally nodulated dual-root soybeans. The dual-root soybeans and nitrogen-free nutrient solutions were prepared according to a method described by Xia et al. (2017) [31]. Details of the grafting process, the composition of the nitrogen-free nutrient solution and the method of rhizobial inoculation are given in the Supplementary Material (Figures S1 and S2 and Table S1). Nitrogen-containing nutrient solution was prepared by the addition of KNO_3_. At the onset of the nitrate supply treatments, K^+^ was supplemented via K_2_SO_4_ in the nitrogen-free nutrient solution.

Before the VC stage, the plants were watered with distilled water once a day. At the VC to V4 stage, the plants were watered with the nutrient solution once a day. From the V4 stage to the end of the experiment, the plants were watered with the nutrient solution twice a day, in the morning and evening. A total of 250 mL of nutrient solution was supplied on each side of the root system. Soybean growth stages were designated as described by Fehr et al. (1971) [32]. During the VC to V4 stage, nitrogen-containing nutrient solution with a nitrogen concentration of 12.5 mg·L^−1^ was used for the growth of dual-root soybeans. The nitrogen starvation was started from the V4 stage; both side roots of the dual-root soybeans were supplied with nitrogen-free nutrient solution for 10 days.

### 4.2. Experimental Treatments

**Figure 5:**
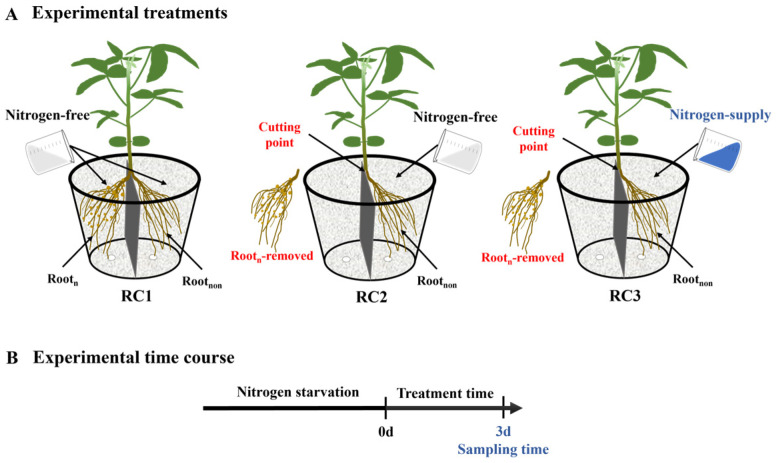
Schematic illustration of the experimental treatments and the experimental time course in experiment I. (**A**) depicts the experimental treatments, while (**B**) outlines the experimental time course. Each pot contains two individual plants. Root_n_, nodulated side roots; Root_non_, non-nodulated side roots.

Experiment I: The effects of nodule nitrogen fixation and nitrate supply on asparagine and ureide content in the shoots of dual-root soybeans were studied in this experiment. After nitrogen starvation treatment, unilaterally nodulated dual-root soybeans were subjected to three different treatments. In the RC1 treatment, both side roots of dual-root soybeans were maintained and supplied with a nitrogen-free nutrient solution. In the RC2 treatment, the nodulated side roots of dual-root soybeans were removed, while the remaining non-nodulated side roots were supplied with a nitrogen-free nutrient solution. In the RC3 treatment, the nodulated side roots of dual-root soybeans were removed, while the remaining non-nodulated side roots were supplied with a nitrogen-containing nutrient solution at a concentration of 200 mg·L^−1^. Figure 5 provides a schematic illustration of the experimental treatments and the experimental time course. All of these experimental treatments were conducted over a period of 3 days. Each treatment was replicated three times, with each replication consisting of one pot, and each pot containing two individual plants. Samples were taken after 3 days of treatment.

**Figure 6:**
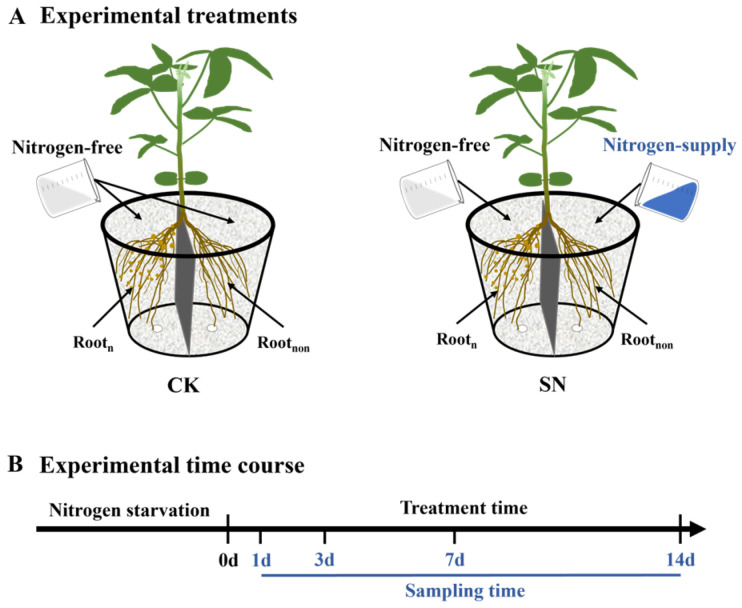
Schematic illustration of the experimental treatments and the experimental time course in experiment II. (**A**) depicts the experimental treatments, while (**B**) outlines the experimental time course. Each pot contains two individual plants. Root_n_, nodulated side roots; Root_non_, non-nodulated side roots.

Experiment II: The dynamic effects of continuous nitrate supply to non-nodulated side roots of dual-root soybeans on nodule nitrogen fixation in nodulated side roots were studied in this experiment. After nitrogen starvation treatment, unilaterally nodulated dual-root soybeans were subjected to two different treatments. In the CK treatment, both side roots of dual-root soybeans were supplied with a nitrogen-free nutrient solution. In the SN treatment, the nodulated side roots were supplied with a nitrogen-free nutrient solution, while the non-nodulated side roots were supplied with a nitrogen-containing nutrient solution at a concentration of 200 mg·L^−1^. Figure 6 provides a schematic illustration of the experimental treatments and the experimental time course. All experimental treatments were conducted over a 14-day period. Each treatment was replicated three times, with each replication consisting of one pot, and each pot containing two individual plants. Samples were taken after 1, 3, 7, and 14 days of treatment.

### 4.3. Sampling Methods

At the time of sampling in experiment I, the soybean plants were cut at the grafting site. The shoots were divided into leaves, petioles, and stems. All samples were washed with distilled water to remove sand from the surface, blotted dry with filter paper, and divided into two groups. In one group, the samples were frozen in liquid nitrogen and then stored in a refrigerator at −80 °C for the determination of asparagine content. In another group, the samples were dried in a constant temperature oven at 60 °C until constant weight and then used for the determination of ureide content.

At the time of sampling in experiment II, the dual-root soybean plants were cut at the grafting site. The shoots were divided into leaves, petioles, and stems. All samples were washed with distilled water to remove sand from the surface, blotted dry with filter paper, and divided into two groups. In one group, the nodules were frozen in liquid nitrogen, and stored in a refrigerator at −80 °C for the determination of urate oxidase activity. In another group, the samples were dried in a constant temperature oven at 60 °C to constant weight and then used for the determination of ureide content. Before drying in a constant temperature oven, the nodulated roots were used for the determination of nitrogenase enzyme activity. After the nitrogenase activity assay, the nodules were removed from the roots and counted.

### 4.4. Analysis Methods

The content of asparagine was determined by the pre-column derivatization method with phenyl isothiocyanate (PITC) [9]. Nitrogenase activity was determined by the acetylene reduction method [31]. The ureide content was determined using a colorimetric method [33]. The urate oxidase activity was determined using colorimetric method [34].

### 4.5. Data Analysis

Data were statistically analyzed using SPSS 21.0 software (IBM, Inc., Armonk, NY, USA). Prior to conducting one-way analysis of variance (ANOVA) and Student’s *t*-test, normality tests were conducted. The correlations between urate oxidase activity with ARA and SNA of soybean nodules were assessed via Pearson’s correlation coefficient.

## 5. Conclusions

Nitrate supply to the non-nodulated side roots of unilaterally nodulated dual-root soybeans systematically inhibited nodulation, nodule nitrogenase activity, and nodule urate oxidase activity of the nodulated side roots. Nitrate supply to the non-nodulated roots enhanced the asparagine content in the shoots of dual-root soybeans, potentially inhibiting ureide catabolism and leading to the accumulation of ureide in the shoots. Ureide accumulation, in turn, caused a decrease in nodule nitrogenase activity through a feedback inhibition pathway. Noduleurate oxidase activity can be used as an important index to assess the nodule nitrogen fixation capacity of soybeans.

## Figures and Tables

**Table 1:** Pearson’s correlation of urate oxidase activity with ARA and SNA of soybean nodules.

Traits	Urate Oxidase Activity
ARA	0.953 **
SNA	0.829 *

Notes: * denoted *p* < 0.05 and ** represents *p* < 0.01.

## Data Availability

All data are included in the main text.

## Supplementary Materials

The following supporting information can be downloaded at: https://www.mdpi.com/article/10.3390/plants13152045/s1, Figure S1: Schematic illustration of cultivating potted unilaterally nodulated dual-root soybeans in sand; Figure S2: Preparation of unilaterally nodulated dual-root soybeans; Table S1: Composition of the nitrogen-free nutrient solution.
