# Supplementary material for: Nitrate Inhibits Nodule Nitrogen Fixation by Accumulating Ureide in Soybean Plants
Source: Plants (Basel). 2024 Jul 25;13(15):2045. doi: 10.3390/plants13152045 (PMC11313793; doi:10.3390/plants13152045)
Supplement: Supplementary file 1 [file plants-13-02045-s001.zip › plants-3052178-supplementary.pdf]

Preparation of the unilaterally nodulated dual-root soybeans and the composition of the nitrogen-free nutrient solution

Soybean plants were grown in sand culture, with two plants per pot (Figure S1). The plastic buckets that were used for the potting were 30 cm in diameter at the top and 28 cm in height. The bucket was divided into two equal parts by a custom-made polycarbonate plastic board, allowing the two roots to grow on either side. The height of the plastic board was 2 cm lower than the bucket. The gap between the board and the bucket was sealed with glue and a 1 cm diameter drain hole was drilled in the bottom of the bucket on each side of the sheet. The sand was rinsed with tap water and then twice with distilled water before filling the buckets. Each bucket contained 20 kg of sand.

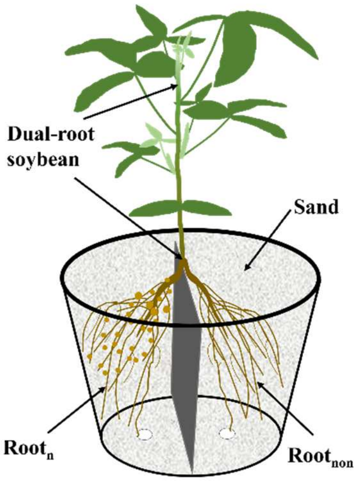

Figure S1. Schematic diagram of cultivating potted dual-root soybeans in sand

Seeds of nodulated soybeans (*Glycine max* L. cv. Heinong 40) and non-nodulated soybeans (*Glycine max* L. cv. WDD01795, L8-4858) were seeded into fine-sand medium and cultured in an illuminated growth chamber at 30°C for approximately 4 days. When the distance between the growing point and the root tip was 7 to 10 cm, the roots of the soybean seedlings were rinsed with water and then used for grafting. First, a sterilised blade was used to make an incision of approximately 0.5-1.0 cm in length in the upper middle of the hypocotyl of the soybean seedling, with the direction of the incision being from down to top in nodulated soybeans and from top to down in non-nodulated soybeans, the non-nodulated roots noted as root$_{non}$, and the nodulated roots noted as root$_n$ (Figure S2A). The two seedlings were cross-inserted into the incisions (Figure S2B) and clipped with a plastic grafting clamp (Figure S2C). The roots of the two seedlings were planted in sand medium in each half of the pot divided by the partition, with the grafting site just above the partition (Figure S2D). After one week, the grafting clamps were removed and the shoot of the non-nodulated seedling was cut off from the grafting site so that the seedling became a whole with two roots sharing the nodulated shoot (Figure S2E, F).

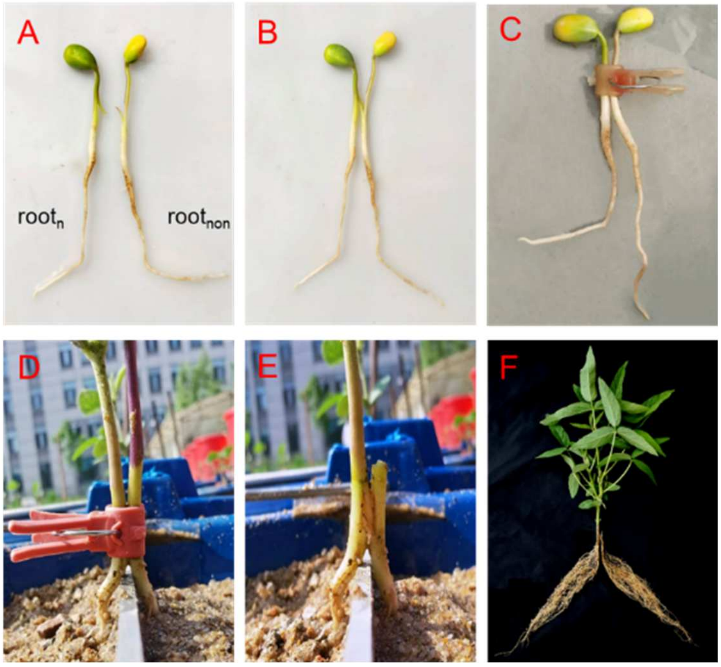

Figure S2. Preparation of unilaterally nodulated dual-root soybeans

Table S1. Composition of the nitrogen-free nutrient solution

Inorganic salts	Concentration (mg·L^{-1})	Inorganic salts	Concentration (mg·L^{-1})
KH_2PO_4	136.00	$ZnSO_4·7H_2O$	0.22
$MgSO_4$	240.00	$MnCl_2·4H_2O$	4.90
$CaCl_2$	220.00	H_3BO_3	2.86
$Na_2MoO_4·H_2O$	0.03	Fe–EDTA*	
$CuSO_4·5H_2O$	0.08		

*Note: The solution which contain 5.57 g $FeSO_4·7H_2O$ and 7.45 g Na_2EDTA per one litre respectively were added into nutrient medium as the rate of 1:1000 when using.
